# Parallel Radars: From Digital Twins to Digital Intelligence for Smart Radar Systems

**DOI:** 10.3390/s22249930

**Published:** 2022-12-16

**Authors:** Yuhang Liu, Yu Shen, Lili Fan, Yonglin Tian, Yunfeng Ai, Bin Tian, Zhongmin Liu, Fei-Yue Wang

**Affiliations:** 1 The State Key Laboratory for Management and Control of Complex Systems, Institute of Automation, Chinese Academy of Sciences, Beijing 100190, China; 2 School of Artificial Intelligence, University of Chinese Academy of Sciences, Beijing 100049, China; 3 School of Information and Electronics, Beijing Institute of Technology, Beijing 100190, China; 4 North Automatic Control Technology Institute, Taiyuan 030006, China; 5 Macao Institute of Systems Engineering, Macau University of Science and Technology, Macao 999078, China; 6 Beijing Engineering Research Center of Intelligent Systems and Technology, Institute of Automation, Chinese Academy of Sciences, Beijing 100190, China

**Keywords:** parallel radars, ACP method, cyber-physical-social systems (CPSS), federated radars

## Abstract

Radar is widely employed in many applications, especially in autonomous driving. At present, radars are only designed as simple data collectors, and they are unable to meet new requirements for real-time and intelligent information processing as environmental complexity increases. It is inevitable that smart radar systems will need to be developed to deal with these challenges and digital twins in cyber-physical systems (CPS) have proven to be effective tools in many aspects. However, human involvement is closely related to radar technology and plays an important role in the operation and management of radars; thus, digital twins’ radars in CPS are insufficient to realize smart radar systems due to the inadequate consideration of human factors. ACP-based parallel intelligence in cyber-physical-social systems (CPSS) is used to construct a novel framework for smart radars, called Parallel Radars. A Parallel Radar consists of three main parts: a Descriptive Radar for constructing artificial radar systems in cyberspace, a Predictive Radar for conducting computational experiments with artificial systems, and a Prescriptive Radar for providing prescriptive control to both physical and artificial radars to complete parallel execution. To connect silos of data and protect data privacy, federated radars are proposed. Additionally, taking mines as an example, the application of Parallel Radars in autonomous driving is discussed in detail, and various experiments have been conducted to demonstrate the effectiveness of Parallel Radars.

## 1. Introduction

A radar is a kind of active sensor that plays an increasingly important role in many fields, such as national defense [1,2], traditional industry [3,4,5,6,7,8,9,10], and autonomous driving [11,12]. Current radars collect data using electromagnetic waves to acquire three-dimensional information about their surroundings. However, due to the increased complexity of the environment, traditional radars are unable to satisfy the needs for new functional requirements, and smart radar systems must be built to adapt to the changing environment in real time. With the rapid development of artificial intelligence and computer science, digital twins in cyber-physical systems (CPS) [13,14,15], which are regarded as the key to the next industrial revolution, are being used to construct digital radars in cyberspace to achieve intelligence. Radar models in CPS [16,17,18,19,20,21,22,23,24,25] have already been extensively researched and demonstrated to be effective in solving many problems, including generating virtual data for various downstream tasks [26,27,28,29,30,31,32,33,34] and closed-loop testing.

In a real application, human involvement exerts a tremendous influence on the operation and maintenance of radar systems; thus, radar systems should be regarded as Morton systems with self-actualization rather than Newton systems [35]. Due to an incomplete consideration of human factors, digital twins radars in CPS are insufficient for the construction of smart radar systems. ACP-based parallel intelligence proposed by Prof. Wang has demonstrated obvious advantages in realizing intelligence in cyber-physical-social systems (CPSS) [36,37,38]. The ACP method comprises artificial societies, computational experiments, and parallel execution, which has already been widely used in many applications, such as control and management [39,40,41], driving [42,43,44], scenarios engineering [45,46], and light fields [47,48,49]. Based on parallel theory, this paper proposes Parallel Radars—a new framework that consists of Descriptive Radars, Predictive Radars, and Prescriptive Radars. This is a paradigm of future smart radars that can not only overcome radar hardware limitations through cloud computing but also make intelligent adjustments in real time. The main contributions of this paper can be summarized as follows:

- To overcome the hardware limitations of radars, we propose the novel framework Parallel Radars, a virtual-real interactive radar system in CPSS. It constructs a complete closed loop between physical space and cyberspace to achieve digital intelligence.
- In order to utilize Parallel Radars’ data more efficiently, federated radars are put forward to connect data silos and protect data privacy.

This paper is organized as follows. Section 2 introduces the principles and applications of commonly used radars briefly. Section 3 presents the framework—Parallel Radars—in detail and federated radars that focus on data security are discussed in Section 4. The experiments about Parallel Radars’ application in mine autonomous driving are desrcibed and analyzed in Section 5. Section 6 presents the conclusions and summarizes the prospects for the future work of Parallel Radars.

## 2. Radar Systems

Radar is a broad concept; there are various types of radars for different applications, such as millimeter wave (mm-wave) radars, LiDARs, and synthetic aperture radars (SAR). In this section, we only focus on mm-wave radars and LiDARs, which are widely applied in autonomous driving.

### 2.1. Principles

In this part, the working principles of mm-wave radars and LiDARs are clarified in detail. Due to the differences in working principles, they have respective characteristics and are deployed for different tasks.

#### 2.1.1. Mm-Wave Radars

Mm-wave radars use electromagnetic waves to measure the range, Doppler velocity, and azimuth angle of the target. The two most frequently used frequencies in automotive mm-wave radars are 24 GHz and 77 GHz, but there is generally a preference for 77 GHz because of its advantages in higher-resolution and smaller antennas [50].

The mode of waveform modulation plays an important role in radar systems. Among all the modulation modes, frequency modulated continuous waveform (FMCW) is the most commonly used method. The transmitted and received FMCW signals, which are also called chirps, can be formulated as (1) and (2); f stands for frequency and ϕ is the phase of the signal.

(1) $$x_{T}=sin\left(2\pi f_{T}t+\phi_{T}\right)$$

(2) $$x_{R}=sin\left(2\pi f_{R}t+\phi_{R}\right)$$

**Figure 1:**
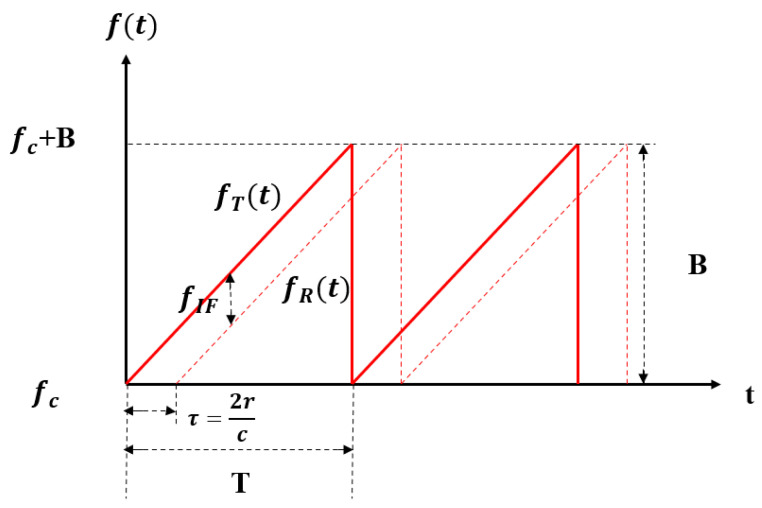
The chirps of FMCW mm-wave radars.

As shown in Figure 1, the frequency of FMCW signals varies linearly in each period, with important parameters including the carrier frequency $f_{c}$, bandwidth B, and signal cycle T. Through the frequency mixer, an intermediate frequency (IF) signal is generated in (3) and beat frequency $f_{IF}$ can be calculated by the frequency of transmitted signal $f_{T}$ and received signal $f_{R}$ in (4). From Figure 1, we can obtain the correlation between $f_{IF}$ and distance *r*, as shown in (5).

(3) $$x_{IF}=sin\left[2\pi \left(f_{T}-f_{R}\right)t+\left(\phi_{T}-\phi_{R}\right)\right]=sin\left[2\pi f_{IF}t+\phi_{IF}\right)]$$

(4) $$f_{IF}=f_{T}\left(t\right)-f_{R}\left(t\right)=\tau \frac{B}{T}=\frac{2r}{c}\frac{B}{T}$$

(5) $$r=\frac{cTf_{IF}}{2B}$$

The information on Doppler velocity requires multiple adjacent IF signals and it focuses on the phase information of signals. Δϕ denotes the phase shift between adjacent IF signals, λ is the wavelength of chirps, and c is the speed of light. The Doppler velocity of the specific target can be calculated using (6) and (7).

(6) $$\Delta \phi =\phi_{IF1}-\phi_{IF2}=\frac{2\pi c(\tau_{1}-\tau_{2})}{\lambda }=\frac{4\pi (r_{1}-r_{2})}{\lambda }=\frac{4\pi vT}{\lambda }$$

(7) $$v=\frac{\Delta \phi \lambda }{4\pi T}$$

**Figure 2:**
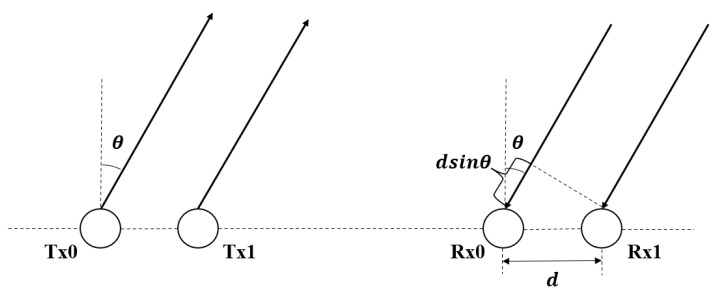
Linear distributed transmitters and receivers in MIMO radars.

In addition, the azimuth angle of the target is calculated based on Multiple Input Multiple Output (MIMO) principles [51] in Figure 2. The distance between adjacent receivers is *d* and IF signals’ phase shift of adjacent receivers ΔΦ. Based on the above information, the azimuth angle of the target θ can be obtained by Equations (8)–(10).

(8) Δr=dsinθ

(9) $$\Delta \Phi =\Phi_{IF1}-\Phi_{IF2}=\frac{2\pi \Delta r}{\lambda }=\frac{2\pi d\sin\theta }{\lambda }$$

(10) $$\theta =arcsin(\frac{\Delta \Phi \lambda }{2\pi d})$$

#### 2.1.2. LiDARs

LiDARs, which are also called laser radars, play an important role in autonomous driving. Instead of emitting electromagnetic waves such as mm-wave radars, LiDARs use laser beams used fpr detection and ranging. The most frequently used wavelengths are 905 nm and 1550 nm in automotive LiDARs. Due to the great collimation of laser beams, LiDARs possess the advantages of a high angular resolution and distance resolution, as well as strong anti-interference ability compared with traditional radars. However, LiDAR’s performance degrades seriously in adverse weather [52,53].

On the basis of the waveform modulation mode, the working principles of LiDARs can be divided into two categories: FMCW and time of flight (ToF). FMCW LiDARs use frequency modulated continuous waves to measure the distance and velocity like FMCW mm-wave radars, while ToF LiDARs play a dominant role in autonomous driving and the range *d* is calculated by the flight time of pulsed lasers Δt directly in (11).

(11) $$d=\frac{c\Delta t}{2}$$

### 2.2. Applications

At present, radars are widely applied in various scenarios, especially in autonomous driving. In this part, the applications of mm-wave radars and LiDARs will be introduced in detail.

#### 2.2.1. Mm-Wave Radars

Mm-wave radars are used for a variety of detection targets, including objects in industry and human beings in biomedical applications [54]. mm-wave radars can not only monitor and measure products in traditional industries, but they can also complete the perception of obstacles in autonomous driving. In the context of biomedical applications, MIMO mm-wave radars enable real-time detection of vital signs including fall detection [55], sleep monitoring [56], and hand gesture recognition [57]. In addition to observing conventional vital signs, mm-wave radars can also be employed as an aid for special populations. For example, Refs. [58,59] introduce a cane with mm-wave radars to help blind people to perceive obstacles, which can facilitate their lives effectively.

Due to the good penetration in rain and snow, automotive mm-wave radars are able to work in all weather conditions. According to the different detection ranges, the current automotive mm-wave radars can be divided into three categories—long-range radars (LRR), middle-range radars (MRR), and short-range radars (SRR)—as shown in Table 1. In real applications, LRR are mainly applied for adaptive cruise control and forward collision warning, MRR for blind spot detection and lane change assistance, and SRR for parking assistance [50]. With the rapid development of radar technologies, the emerging 4D mm-wave radars can potentially replace automotive LiDARs in the future.

#### 2.2.2. LiDARs

LiDARs are able to provide dense point clouds of surroundings. Due to the obvious advantages in accuracy, LiDARs are suitable for various high-precision tasks and can be classified into three categories according to different platforms in Table 2. Spaceborne LiDARs are mainly used for space rendezvous, docking, and aircraft navigation [60]. Airborne LiDARs are designed for the tasks such as terrain mapping and underwater detection [61], while vehicle-borne LiDARs play an important role in autonomous driving.

### 2.3. Future of the Radar Industry: From CPS to CPSS

With the development of computer science, digital twins’ radar systems in CPS have received extensive attention and shown great advantages in cost. However, they not only ignore the interaction between the physical and virtual world but also oversimplify various important factors in physical scenarios. Additionally, the current digital twin radar systems also neglect human factors in social space, which are closely related to each part of radar systems. Human dynamics introduce predictive uncertainty into radar systems, which is a great challenge to the framework of digital twins’ radars. In order to solve the above problems and achieve digital intelligence, Parallel Radars in CPSS that are tightly coupled with physical space, cyber space, and social space are proposed as a novel method framework. A comparison of digital twins’ radars and Parallel Radars is shown in Table 3. Parallel Radars construct complete artificial systems in cyber space, provide a mechanism to realize knowledge automation through computational experiments, and conduct intelligent interactions between physical and digital radars.

## 3. Parallel Radars

**Figure 3:**
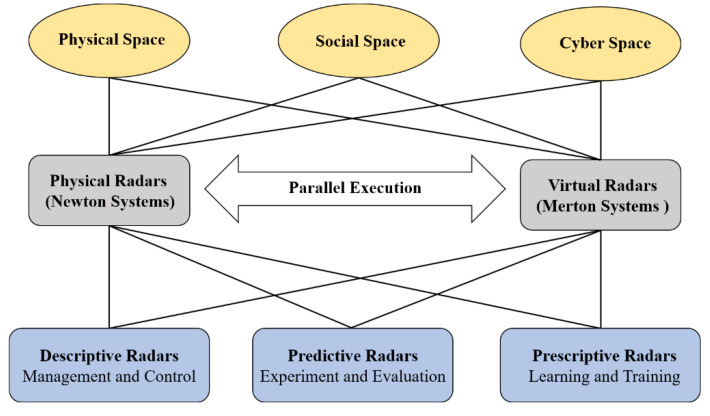
The framework of Parallel Radars.

On the basis of parallel intelligence and ACP method [36], Parallel Radars, a new technical framework leading the future development of radar systems in Figure 3, is proposed. Parallel Radars integrate traditional radar knowledge with artificial intelligence and advanced cloud computing, as well as 5G communication technologies. It is able to provide efficient, convergent solutions to achieve smart radar systems in CPSS through data-driven Descriptive Radars, experiment-driven Predictive Radars, and Prescriptive Radars for interaction between the physical and virtual radars.

Descriptive Radars construct artificial systems for describing radars in cyber space and can be used to collect large amounts of virtual data. Predictive Radars conduct various computational experiments with artificial systems and generate deep knowledge, while Prescriptive Radars take feedback control of both physical and artificial systems and complete parallel execution. In the following subsections, we take autonomous driving as an example to introduce the framework of Parallel Radars in detail.

### 3.1. Descriptive Radars

**Figure 4:**
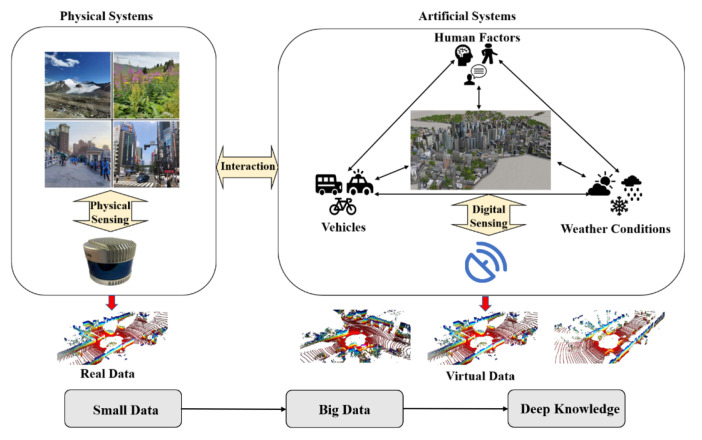
Descriptive Radars: the framework and process.

Traditional radar sensors only complete the data collection without the ability to adjust operating modes in real time. Useless redundant data will be collected and only can be processed with local computing devices that drop radars’ performance seriously. Descriptive Radars that correspond to artificial radar systems in ACP method are proposed for better operation and management of radar systems, as shown in Figure 4. Each descriptive radar can be viewed as the mirror image of a physical radar in artificial systems. Apart from high-fidelity radar models, Descriptive Radars are closely related to scenario engineering and it’s the pioneering work to take the social environment into consideration. The physical environment in artificial scenarios includes common buildings, driving cars, and different weather conditions, while the social environment focuses on human behaviors and knowledge. Due to the involvement of social space, Descriptive Radars are able to generate massive more realistic synthetic data compared with the current digital twins’ radars in CPS [16,17,18,19,20,21,22,23,24,25].

Descriptive Radars should build complete artificial systems in the cloud at first. In terms of sensor modeling, advanced ray tracing technology [62,63] is able to imitate the process of data collection and AutoCAD [64] for modeling radars’ physical structures precisely. The working conditions of Descriptive Radars should be consistent with physical radars to ensure the fidelity of virtual data. The developed game engines such as Unreal Engine [65] and the industrial engines Omniverse [66] can be applied to build artificial scenarios, while the novel 3D reconstruction technique, Nerf [67], is also a suitable method for environment modeling. Synthetic data can be collected by moving the sensor models in artificial traffic scenes. Compared with end-to-end generation models, such as GANs [68] and diffusion models [69], Descriptive Radars have better interpretability and are able to obtain labels directly. In a real application, Descriptive Radars are responsible for modifying the incremental information that can reduce the process of redundant data and break the physical limits of hardware through cloud computing. They also can generate big virtual data at a low cost and provide security for radar systems when physical radars fail. To be specific, virtual data are mainly used for augmenting real training sets to enhance the model performance. We use a large amount of virtual data for models pre-training and conduct fine tuning with small real data. The generated virtual data have alredy been proven effective for object detection [26,27], segmentation [28,29,30,31], and mapping [32,33,34]. Additionally, Descriptive Radars can also be used to extract the hidden features of different traffic scenes to make the model achieve better generalization.

### 3.2. Predictive Radars

**Figure 5:**
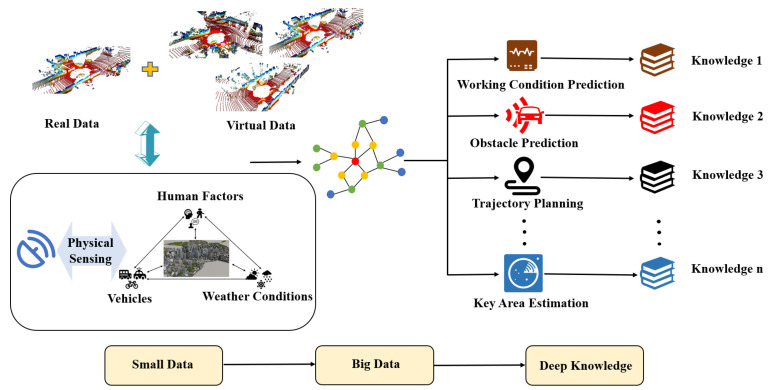
Predictive Radars: the framework and process.

Although Descriptive Radars have already constructed artificial systems in cyber space, there is an obvious problem that automotive radars are facing in a dynamic environment in practice. Due to human involvement, the static scenes established by Descriptive Radars are impossible to cover all possible scenes of the physical world. It is challenging to achieve the optimal scheme and provide help for physical radars. Predictive Radars, as shown in Figure 5, are proposed for experiments and evaluation with artificial systems to solve this problem. They are similar to the process of imagining the consequences in mind before making a decision. Predictive Radars conduct various computational experiments with artificial systems, which is also the transition process from small data to big data to deep knowledge. Data, including real data and virtual data, are used to achieve deep knowledge that can be understood by humans clearly.

Predictive Radars take small data of the current scenes as input and perform general perception tasks such as PointPillars [70] for object detection and PointNet++ [71] for segmentation at first. The emerging cooperative perception [72,73,74,75] can greatly increase the perception range of each vehicle and deserves more attention in the future. Based on the results of general perception tasks, Predictive Radars will predict different future scenes which is the transition from small data to big data. It is essential to apply trajectory planning technology [76,77,78,79,80] to conduct predictions while the network structures of Transformer [81,82] and N-Bests [83] have shown great advantages and show promise for wide application. After acquiring data for future scenarios, we should aggregate features in the time axis, evaluate different situations, and find out the optimal strategy to develop deep knowledge from big data. Predictive Radars are able to be applied to many specific problems, including working conditions prediction, obstacle prediction, and key area estimation. It can increase the utilization efficiency of hardware resources and avoid the process of redundant data.

### 3.3. Prescriptive Radars

**Figure 6:**
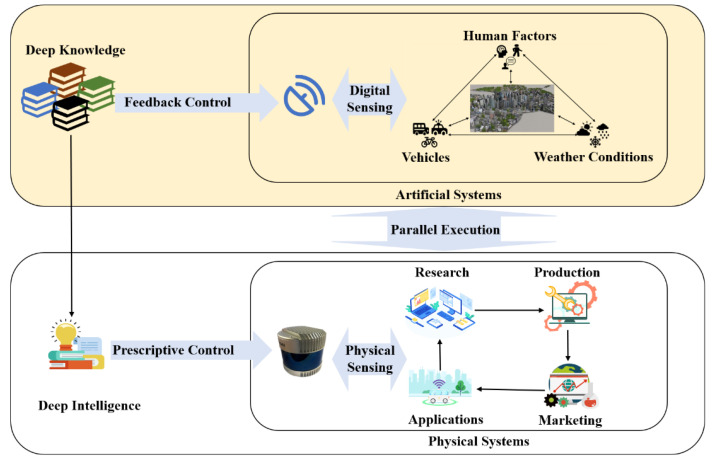
Prescriptive Radars: the framework and process.

At present, radars are only used to collect data in the fixed operating mode and there is no interaction between the physical radars’ hardware and generated deep knowledge through computational experiments. However, automotive radars are facing a dynamic and complex environment in CPSS with human involvement during application. It is essential to build smart radar systems to meet new requirements for intelligent information processing in real time. With the assistance of Descriptive and Predictive Radars, deep knowledge has already been achieved through various computational experiments while Prescriptive Radars in Figure 6 are proposed to take prescriptive control of physical radars and complete parallel execution. Prescriptive Radars use software to redefine radar systems as interactive systems tightly coupled between physical and virtual radars instead of regarding them as two isolated systems.

On the basis of obtained deep knowledge in cyber space, Prescriptive Radars provide feedback to physical radars and adjust operating modes in real time with the assistance of digital technologies [84]. Novel modulation methods such as OFDM [85] and PMCW [86] can generate waveforms digitally with timely adjustment and they have shown great flexibility and intelligence in radar systems. Prescriptive Radars can change the waveforms’ type and sparsity of beams to focus on key areas that help radar systems adapt to different weather conditions and emergencies. In the real application, Prescriptive Radars keep virtual radars consistent with physical radars all the time to conduct parallel execution and constituted a complete closed loop.

## 4. Federated Radars

Parallel Radars can realize data fusion through the network of multiple radars, which has not only constructive benefits for perception tasks but also lower requirements for communication bandwidth with cost savings.

The improvement of algorithms has accelerated the development of big data and hardware computing power. However, the existing centralized training mode makes the deployment of Parallel Radars face many difficulties and challenges. It is mainly reflected in the high privacy of data collected by each radar system. These sensitive data are forbidden to be uploaded without authorization so that the data of each client can’t be shared. However, the effect of deep learning models depends on the quantity and quality of data. Limited by the data fragmentation, the models are only fed with local data for single-point modeling, which affects the model performance seriously. In addition, due to the differences in device performance, data synchronization is inconsistent among various data sources and the current scene cannot be responded to in real time. Non-uniformity in data distribution, quality, and size also affects model performance seriously.

**Figure 7:**
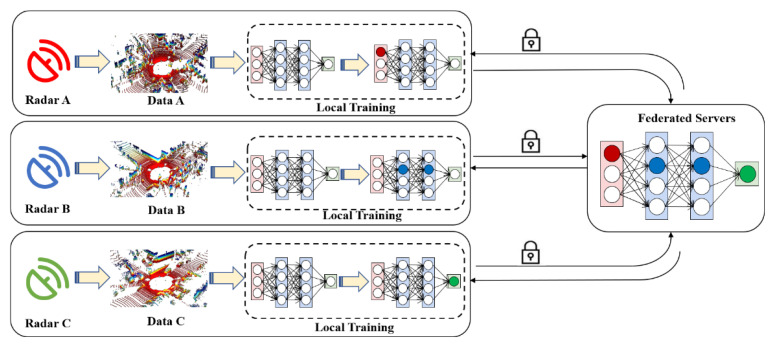
Federated Radars: The Framework and Process.

Data privacy threats including data theft and data leakage after leaving the local area need to be solved urgently [87], especially for military use. As a method to connect the silos of data, federated learning [88] enables participants to build models without sharing data to achieve swarm intelligence [89]. Under this framework, federated radars in Figure 7 use common data to train a model for multiple radars [90], while the federation of radars can realize model interactive and cooperative learning between different radars [91]. In federation of radars, data are aggregated in each client for local training. The models will be deployed locally and uploaded to the cloud servers after local training. When the performance of global models in the cloud exceeds the original local models, they can be deployed to other clients with permission. They can work across different data structures and different institutions. It has advantages in lossless model quality and data security without the limitation of algorithms [92]. Each radar system based on federated learning can collect and process data independently, and is entitled to initiate federated learning that speeds up the deployment of new models for radars. During the process of model uploading, the transmission volume of model parameters is much smaller than the transmission of data, which can save network bandwidth effectively [93].

## 5. Applications of Parallel Radars

Parallel Radars in CPSS realize digital intelligence with virtual–real interactions and can be widely applied in many fields. In this section, we focus on three main application cases of Parallel Radars, including burgeoning autonomous driving, traditional industry, and military use. In autonomous driving, we take mines as an example to introduce the technical implementation of Parallel Radars and conduct various experiments.

### 5.1. Autonomous Driving

Parallel Radars play an important role in autonomous driving. The high cost of collecting data and long-tail problem are serious issues. With the complete artificial systems in cyber space, Parallel Radars can generate sufficient virtual data to train new models for different downstream tasks such as object detection [26,27], segmentation [28,29,30,31], and cooperative perception [72,73,74,75], which can solve these problems effectively. Specific tasks such as the validation of new radars [94], super-resolution [95,96,97], and the analysis of radar placement [98,99], can also be settled. Additionally, due to the limitation of local computing resources, it is impossible to conduct various predictive experiments locally. Parallel Radars can help solve prediction problems in the time domain effectively such as trajectory planning, working condition prediction, and critical area estimation through computational experiments in the cloud. They are able to provide instructions for the next movement of radars and avoid redundant data processing. Parallel Radars use software to redefine radar systems and are able to take indicative control of radar’s hardware based on the obtained deep knowledge in real time. Parallel execution can be conducted with the growing digital technologies such as digital beam forming and digital waveform generation [85,86]. In addition to the application process, Parallel Radars can also take prescriptive control of each step of the radar industry, including research and manufacturing, as well as marketing.

Due to the complex environment, it is difficult to realize autonomous driving in urban areas in a short time, while specific scenes such as mines and ports are promising to make autopilot apply in the first place. On the basis of parallel intelligence, we apply Parallel Radars to mines and carry out dozens of experiments.

**Figure 8:**
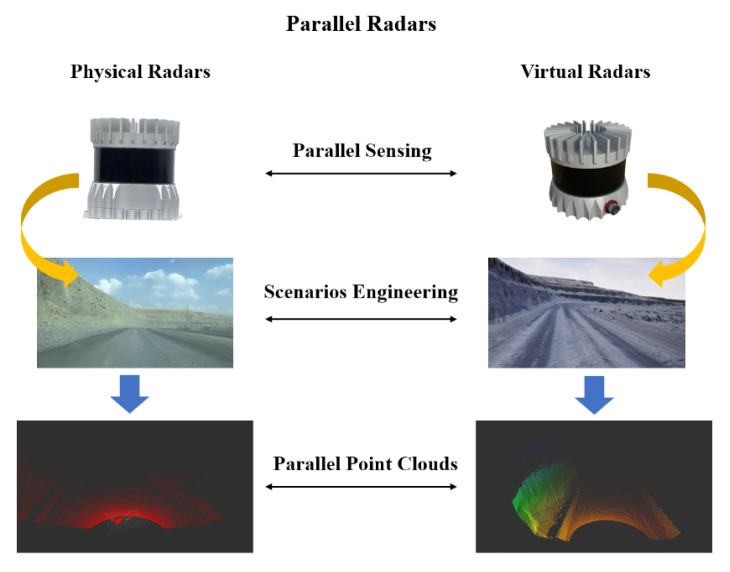
Application of Parallel Radars in mines.

First, as shown in Figure 8, we build artificial radar systems including sensor models and scenarios of mining areas based on Unreal Engine 4 to generate virtual data in cyber space [100]. In our artificial systems, virtual radars are consistent with real radars in appearance and internal physical parameters. It can not only collect a lot of data through artificial mines, which are costly in physical space, but also maintain safe operation when physical radars fail.

**Figure 9:**
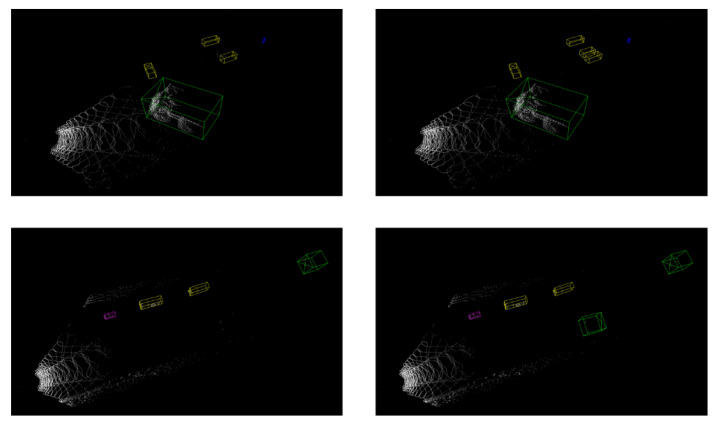
Comparative experiments between the baseline model and new model in object detection. The left column is the baseline model trained only on real data and the right column is the new model trained on both real data and virtual data.

Second, we conduct computational experiments on object detection to verify the effectiveness of collected virtual data. We only utilize real mining data to train a PointPillars model as the baseline and mixed data including real and synthetic data is applied to train a new model. Both models are tested in real testing set for qualitative analysis. We find that the additional virtual training data can improve models’ performance effectively as shown in Figure 9. Vehicles in the distance with only a few points are missed by the baseline model while they can be successfully identified and localized by the new model.

**Figure 10:**
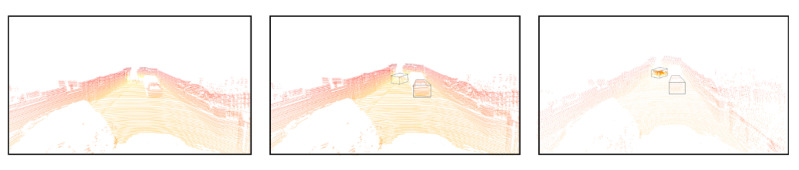
Critical area prediction and scene rescan based on Parallel Radars.

Finally, we conducted experiments to realize the prescriptive control of Parallel Radars. The left picture in Figure 10 is a frame of point cloud data collected in the normal operating mode and the middle one highlights the detected bounding boxes. We found that a vehicle with only a few points is changing lines in the distance while it is important for subsequent decisions and requires more attention. On the basis of perception results, we put more hardware resources on the estimated critical areas in order to obtain locally dense point clouds as shown in the right picture of Figure 10. More detailed information on key areas can be achieved through Parallel Radars. It realizes the feedback from obtained deep knowledge through computational experiments in cyber space to physical radars and builds a complete closed loop.

Taking mines as an example, we introduce the working process of Parallel Radars in autonomous driving and provide concrete methods at the technical level. In the future, we will apply Parallel Radars to predict the occurrence of landslides which is a common but dangerous phenomenon in mines.

### 5.2. Traditional Industry

Parallel Radars can also be widely applied in traditional industries, such as architectural design [3], observation [4,5,6,7], industrial robots [8], and heating, ventilation, and air condition (HVAC) control [9,10]. In the field of architectural design, Parallel Radars are helpful in the 3D modeling and analysis of architectures with the consideration of human factors. It can predict the evolution of a building from its establishment realistically at short notice. For observation tasks, Parallel Radars can be employed in the observation of burden surface inside blast furnaces [4], the concentration of air pollutants [5], and the structural failure of wind turbines [6,7]. They can predict potential problems in advance and lead physical radars to pay more attention to critical areas. It ensures the security of equipment while utilizing resources efficiently. Parallel Radars also play an important role in industrial robots that enable the precise positioning of industrial robots at a low cost. Additionally, Parallel Radars can realize intelligent control in HVAC systems, which can reduce energy consumption effectively. With a complete consideration of human factors, parallel radars allow for accurate monitoring and prediction of human behaviors.

### 5.3. Military Use

Parallel Radars also play an important role in military use which is the initial application scenario of radars [84,101]. There are several radar systems used in the military such as early warning radars for target sensing in the distance and tracing radars for target tracking. Parallel Radars can provide predictive information through computational experiments with artificial systems. It can not only help radar systems discover potential targets earlier but also be used for robustness testing of tracking algorithms. Additionally, due to the requirement of a large survey region, military radar systems take a huge energy consumption. Parallel Radars are able to take intelligent adjustments of operating modes according to the environment to reduce energy consumption.

## 6. Conclusions

Traditional radars must evolve into smart radar systems to adapt to the dynamic and complex environment. Due to the neglect of human involvement and virtual–real interactions, digital twin radars in CPS are insufficient to achieve true intelligence. However, parallel intelligence in CPSS can be applied to construct a novel methodology framework of radars. Based on parallel theory and ACP method, we propose Parallel Radars, a new paradigm of future radars for digital intelligence. Parallel Radars follow the principle of “from small data to big data to deep intelligence” and achieve knowledge automation. Taking the case of autonomous driving in mines, the application process of Parallel Radars is clarified and various experiments are conducted for validation. They have boosted the performance of physical radars efficiently and shown great potential in solving more problems. In order to connect data silos and protect data privacy, the concept of federated radars and federation of radars are discussed. The proposed Parallel Radars offer an effective solution for accomplishing smart radar systems and they are convinced to be widely applied in the future.

## Figures and Tables

**Table 1:** Comparison of different automotive mm-wave radars [50].

	LRR	MRR	SRR
Detection Range (m)	10–250	1–100	0.15–30
Azimuth Field of View (${}^{\circ }$)	20	80	160
Elevation Field of View (${}^{\circ }$)	10	10	20
Range Resolution (m)	0.1–0.5	0.1–0.5	0.02–0.1
Velocity Resolution (m/s)	0.6	0.6	0.6

**Table 2:** Comparison of LiDARs on different platforms.

	Spaceborne LiDARs	Airborne LiDARs	Vehicle-Borne LiDARs
Wavelength (nm)	532/1064	532/1064	905/1550
Detection Range (km)	400	3–6	0.2–0.3
Range Accuracy (cm)	10–50	10–25	1–2
Azimuth Field of View (${}^{\circ }$)	120	360	360
Elevation Field of View (${}^{\circ }$)	120	75	40
Horizontal/Vertical Resolution	Low	Middle	High

**Table 3:** Comparison between digital twins’ radars and Parallel Radars.

	Digital Twins’ Radars	Parallel Radars
Sensor Models	✓	✓
Physical Scenarios	✓	✓
Social Scenarios	×	✓
Virtual-Reality Interaction	×	✓

## Data Availability

Data not available due to commercial restrictions.

## Abbreviations

The following abbreviations are used in this manuscript:

CPSS	Cyber-Physical-Social Systems
CPS	Cyber-Physical Systems
mm-wave radar	millimeter wave radar
LiDAR	Light Detection and Ranging
SAR	Synthetic Aperture Radars
FMCW	Frequency Modulated Continuous Waveform
IF	Intermediate Frequency
MIMO	Multiple Input Multiple Output
ToF	Time of Flight
LRR	Long-Range Radars
MRR	Middle-Range Radars
SRR	Short-Range Radars
HVAC	Heating, Ventilation, and Air Condition
